# Quarterly Report of Proceedings

**Published:** 1857-04

**Authors:** 


### QUARTERLY REPORT OF THE PROCEEDINGS.

Monday, March 2, 1857.

### PRESENTATIONS.

The following donations to the Library of the Society were announced:—

1. On the Treatment of Cholera. By Henry Jeanneret, M.D. Presented by the Author.

2. Die Ruhr nach ihrem endemischen und epidemischen Vorkommen, von ätiologisch-pathologischen Standpunkte geschildert, von Dr. August Hirsch in Danzig.

3. Monatsblatt für Medicinische Statistik und öffentliche Gesundheitspflege, Nos. 4, 5, 6, 7, 8, 9, 10, 11. Beilage zur Deutschen Klinik. Herausgegeben von Dr. Göschen und Dr. S. Neumann.

The above German works were presented by their respective authors through Dr. Weber, Foreign Secretary for Germany and Russia.

4. Essai de Mortalité comparée, avant et depuis l'introduction de la Vaccine en France, par H. Carnot, ancien Officer d'Artillerie, Membre de Legion d'Honneur. Presented by the Author.

### NEW MEMBERS.

*Non-resident.*—Geo. W. Spence, M.D. of Lerwick, Shetland.

John N. Radcliffe, M.R.C.S. of Bramley, Leeds, was proposed as a non-resident member.

### NEW OFFICERS.

The President, Vice-Presidents, the Council, the Treasurer, and the Honorary Secretaries and Foreign and Colonial Secretaries for 1857-8 were proposed for election at the meeting in April.

### PAPER READ.

"On Puerperal Fever" (concluded). By E. W. Murphy, M.D. The paper was discussed by Dr. Tripe, Dr. Chowne, Mr. Hunt, and Dr. Milroy.

